# Necrosis of *Staphylococcus aureus* by the Electrospun Fe- and Ag-Doped TiO_2_ Nanofibers

**DOI:** 10.5402/2012/763806

**Published:** 2012-09-18

**Authors:** Asem Aboelzahab, Abdul-Majeed Azad, Vijay Goel

**Affiliations:** ^1^Department of Bioengineering, The University of Toledo, Toledo, OH 435606-3390, USA; ^2^Department of Chemical Engineering, The University of Toledo, Toledo, OH 435606-3390, USA; ^3^Department of Orthopedic Surgery, The University of Toledo, Toledo, OH 435606-3390, USA

## Abstract

Postsurgery infections cause prolonged hospitalization, incurring increased patient and hospital costs, making it increasingly vital to develop an effective solution for the mitigation and elimination of infection buildup at these sites. Incorporation of a bactericidal device at the infection-prone sites provides the capability of attacking bacterial growth even after the patient has left the hospital. Polycrystalline titanium dioxide (TiO_2_) is photoactive and possesses antibacterial properties that can mitigate the onset of these infections and aid in wound healing. In this work, TiO_2_ nanofibers were synthesized by electrospinning. Doping with iron as well as with silver (5 wt% and 1 wt%, resp.) was also carried out to increase their effectiveness towards bactericidal properties. The electrospun fibers were processed and tested in the presence of light in the suspensions of methicillin-susceptible *Staphylococcus aureus* (MSSA) bacteria, which are the leading infection-inducing bacteria among hospital patients. It was found that upon brief activation (cf. 30 s) by an infrared laser source, greater than 90% of the *S. aureus* was rendered inactive within cf. 10 min. of exposure, thereby showing the potential of titania nanofibers for effective mitigation of infection.

## 1. Introduction

Infections caused by bacteria could pose harmful attacks on human tissues to a point where fighting them off becomes increasingly difficult. Though the source and reasons of infection and propagation are multifarious, the consequences could be debilitating and disastrous. In addition to the health risk, the financial costs are also huge. The gram-positive *Staphylococcus aureus* (*S. aureus*) microorganism is the leading cause of infection in healthcare settings where even antibiotic treatment has proven to be exhausted without appreciable effect in many cases [1–4]. One study containing data accumulated over a three-year period of patients admitted to a Boston hospital revealed a mortality rate of 22.9% in patients diagnosed with MSSA and methicillin-resistant *S. aureus* (MRSA) infection, with average lengths of stay between 7 and 9 days and hospital charges between $19,000 and $26,000, respectively [5].

The photocatalytic/antibacterial behavior of titania nanoparticles and their ability to inhibit bacterial proliferation by inducing cell necrosis have been extensively reported [10–14]. Recently, the efficacy of self-standing electrospun TiO_2_ nanofibers and nanofilm coatings on Ti substrates (plates and wires), in initiating the necrosis of *E. coli* cells upon brief activation by infrared radiation, was also demonstrated [15–17]. Others have investigated the propensity of ultraviolet- (UV-) activated TiO_2_ powders and coatings [6–8]; however, in these cases, exposure for much longer duration was necessary to obtain significant cell death. The release of highly active radical oxygen species (ROSs) upon activation of the photoactive TiO_2_ for a longer period could create an unfavorable environment for cell proliferation, thereby limiting the ability of bacteria to multiply and cause further harm to patients with infections [18, 19]. With the use of an IR laser source, high bacterial cell necrosis rate was achieved in much shorter time.

The necrosis ability of many known antibacterial agents has been reported extensively. A large pool of published literature indicates the antibacterial viability of Ag- and Fe-doped materials [20–23]. In addition, it has been reported that Fe- and Cu-modified titania coatings aid in preventing biofilm formation as well as increasing the efficacy of multimodal imaging.

A number of studies showing the antibacterial aspects of nanoscale silver and iron oxide nanoparticles have been reported. For example, Ashkarran [20] showed the effect of silver doping in *E. coli* cell necrosis by titania. The combined effect of increased dopant concentration and the exposure time of illumination by UV or visible light was correlated with titania's antibacterial response. The observed enhancement in the cell necrosis was attributed to the production of radical oxygen species (ROSs), responsible for the deactivation of the enzymes. Page et al. [22] observed increased antibacterial activity with Ag-doped titania coatings compared to undoped ones.

Similarly, Speigling [23] and Foster et al. [24] have, respectively, reported on the antimicrobial activities of Ag-containing titanium alloys and TiO_2_/Ag and TiO_2_/CuO films prepared by CVD process. Finke et al. [25] also observed the antibacterial potential of copper-containing Ti implants. Copper oxide is biocompatible and is benign in smaller concentrations.

The use of supermagnetic iron oxide nanoparticles (SPIONs) in the form maghemite (*γ*-Fe_2_O_3_) to prevent biofilm formation was demonstrated by Taylor and Webster [26] against Zhou et al. [27] against *S. epidermidis*.

Another reason for using silver and iron oxide dopants was to modify the energy bandgap of pure titania (3–3.2 eV) to lower end by causing bandgap bending and creating additional energy states in the vicinity of Fermi energy level by means of more conducting species such as silver and iron oxide [28].

Owing to the frequent occurrence of MRSA-related infections in hospitals and their relevance to the orthopedic post-surgery scenarios resulting in 300,000–500,000 infections in the USA alone each year [29], the present investigation was undertaken to assess the efficacy of the electrospun titania nanofibers towards the necrosis of *S. aureus* when irradiated briefly with IR laser.

## 2. Methods and Materials

### 2.1. Preparation of Polymeric and Titanium Precursors 

The pure and doped titania fibers in nonwoven format were fabricated by electrospinning using an indigenously assembled setup, described in details elsewhere [15, 16]. Titanium (IV) oxynitrate (TiO(NO_3_)_2_) was used as the titanium precursor while water soluble iron (III) nitrate and silver (I) nitrate served as the source chemicals for iron and silver doping, respectively. For the preparation of titanium oxynitrate (TON) solution, 0.6 g of titanium powder (−200 mesh, 99.5%, Alfa Aesar) was dissolved in a mixture of 20 mL HNO_3_ (ACS grade) and 30 mL H_2_O_2_ (ACS grade, 69.5% w/w). The solution was left under constant stirring overnight on a stir plate to form 0.4 M TON. The liquid was filtered into a clean container, discarding the remaining solid. This stock solution was stored at room temperature.

To prepare the 5 wt% Fe-doped TON solution, 100 mg of ferric nitrate (Fe (NO_3_)_3_, Alfa-Aesar, 98+ %) was dissolved in 100 mL of DI water. 50 *μ*L of this solution was added to 5 mL of TON solution made earlier. The solution was thoroughly stirred magnetically to homogenize and stored at room temperature.

The Ag-doped TON solution was made following a similar protocol. A 0.1 M solution of silver nitrate was prepared by dissolving 168.7 mg of AgNO_3_ (Alfa-Aesar, 99.9+ %) in 10 mL of DI water. 290 *μ*L of this was added to 27 mL of TON, stirred thoroughly, and stored at room temperature. This gives spinnable 1 wt % Ag-doped TON solution.

Polyvinylpyrollidone (PVP) with an average molecular weight of 1.3 × 10^6^ Dalton was dissolved slowly in ethanol to obtain its 15 wt% solution. The mixture was heated at approximately 60°C with constant stirring on a heat/stir plate. Once PVP fully dissolved forming a viscous solution, the solution was covered, capped tightly, and stored at room temperature. Due to the pronounced volatility of ethanol during and after the preparation and the tendency of the solution to dry out and leave a stiff gel in the container upon prolonged storage, the PVP solution was prepared in small batches and only when electrospinning was to be carried out.

### 2.2. Electrospinning TN/PVP Solution and Nanofiber Formation 

For electrospinning (e-spinning) experiments, the precursors were mixed in different ratios that were arrived by several optimization trials. The volume/volume (v/v) ratios of 1 : 2 for pure TON + ethanolic PVP solution, 1 : 2 for silver nitrate-TON + PVP and 1 : 3 for iron nitrate-TON + PVP, were found to yield the best results. Respective mixtures were made by mixing the inorganic and organic precursors and manually stirring them with a 1.75 mm stainless steel needle into homogeneous viscous solutions.

Each of the three composite mixtures was drawn into an array of ten 5-mL capacity clinical syringes. Precision-tip 25-gauge stainless steel needles were attached to each syringe, and the entire array was mounted on a programmable syringe pump (KD Scientific model 230, MA, USA). The preferred orientation of the syringe pump in this work was horizontal. A custom-made direct current power supply with a high voltage system (30 kV maximum) described earlier was used for e-spinning [15, 16]. One terminal of the power supply was connected to a metallic wire that looped through all the needles.

For the ease of sample handling and subsequent thermal processing, ceramic plates instead of metallic ones were used as the collector terminal. Moreover, in order to enhance the fiber collection area, a modified collection setup was devised. Two high-density alumina containers (76.2 mm long × 50.8 mm wide × 6.35 mm deep) were employed. The two containers were placed next to each other. Short lengths of electrical wires were attached to the back of each plate at their center by 1-sq. inch blocks of aluminum foil stuck by electrical tape. The other ends of the electrical leads were twisted into a common junction for the collector.

**Figure 1:**
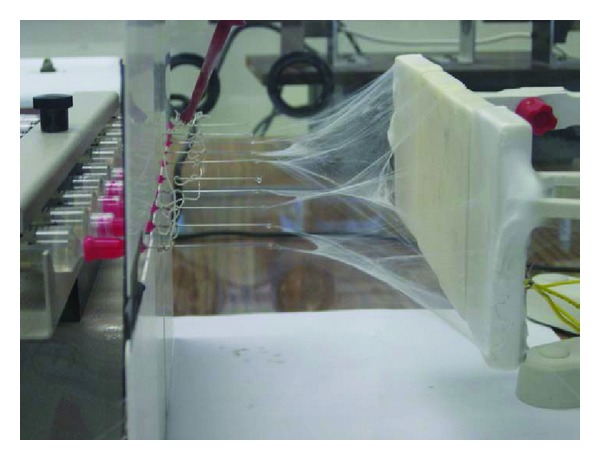
Progress of electrospinning and collection of the non-woven ceramic-polymer (cermer) composite on the ceramic plates.

Using the high-voltage power supply, an electrical impulse was applied between the needle and the collectors in order to initiate the e-spinning. The syringe pump was started and the high-voltage supply unit was turned on. The voltage was tweaked precisely until the fibers began to form steadily and collect on the plates placed 63.5 mm. away from the tip of the needle; the optimized voltage in this case was found to range between 16 and 18 kV. A flow rate of 0.05 mL/h was chosen and found to be adequate. The cermer (ceramic-polymer composite) fibers were spun continuously with short intermittent interruptions of the run for periodic cleaning of the clogged needle tips from time to time.  Figure 1 shows the experimental setup for electrospinning and the non-woven matt of the electrospun cermer composite deposited on the collector plates with time.

### 2.3. Processing and Characterization of the Electrospun Fibers

After spinning was complete, small amounts of the as-spun composite fibers were used for viewing under scanning electron microscope. This allowed one to ensure the quality of fibers in terms of the absence of intertwining, twisting, branching, liquid globule entrapment, and so forth. The remaining fibers collected on the ceramic plates were fired at 700°C for 2 h in static air as per the following heating rate-temperature-soak time profile: 25°C (room temperature) to 500°C at a rate of (1/2)°/min. with a hold at 500°C for 2 h; 500°C to 700°C at a rate of (1/2)°/min. with a hold at 700°C for 2 h, followed by cooling from 700°C to the room temperature at a rate of (1/2)°/min. The rather small heating and cooling rates were chosen so as to ensure the removal of organic components without destroying the nanofibrillar morphological features in the end product and also to avoid the disintegration of the nanofibers into a powdery mass. The final product was pure TiO_2_ in one case and TiO_2_ doped with Ag and Fe_3_O_4_ in the other two.

The fired samples were characterized by a host of techniques, such as, powder X-ray diffractometry (XRD- PANalytical X'Pert Pro MPD), scanning electron microscopy (Hitachi S-4800 UHR SEM), and transmission electron microscopy (Hitachi HD-2300 STEM), both with attachments capable of carrying out energy dispersive spectroscopy (EDS).

### 2.4. Bacterial Growth

For bacterial growth, two subculture flasks with 150 mL of TSB super broth (32 g of tryptone, 20 g of yeast extract, and 5 g of sodium chloride per 1L DI water, Fisher Scientific, Waltham, MA, USA) each was inoculated with 1 mL of *S. aureus* (NRS72, Sanger476; MSSA), which was taken from a previously purified batch culture growth. A standard calibration curve was created with the aid of streaking of cell suspensions on TSB/agar to enumerate cell concentration. The *S. aureus* cells were grown to stationary phase, diluted to a concentration of 9.52 × 10^8^ cells/mL, and then divided into 1 mL aliquots in 3 mL capacity eppendorf tubes for individual experiments. Stock solutions were also stored in 1 mL aliquots with the addition of 30% glycerol for future growth of more bacterial samples. All samples were stored at −80°C until use.

### 2.5. In Situ Image Analysis

For analysis and imaging of cell necrosis of the bacterial suspension during experimentation, an Invitrogen Baclight Live/Dead Assay kit (Invitrogen, Carlsbad, CA, USA) was used in addition to confocal microscopy to obtain real-time imaging of the bacterial cells while in suspension with the calcined fibers, upon being activated by the IR laser. This assay uses SYTO9 and propidium iodide stains, which were stored in 1 *μ*L aliquots for individual experimental use.

100 *μ*L of *S. aureus* suspension were diluted in ultrapure water (with a conductivity of 5.5 × 10^−8^ S-cm) to a 1 : 3 v/v ratio to a volume of 300 *μ*L and cell concentration of 3.17 × 10^8^ cells/mL, with SYTO9 and PI stains added in 1 *μ*L quantities, respectively. The suspension was set in the dark for 20 min. to complete staining. 10 *μ*L of the suspension was then mixed with 30 *μ*L of ultra-pure water (1 : 4 dilution) and added into a 35 × 14 mm glass bottom microwell dish (no. 15 cover glass). This gave a final concentration for imaging of 7.93 × 10^7^ cells/mL.

Approximately 6 mg of the fiber specimen were introduced to the bacterial suspension and activated by a handheld IR laser (**λ** = 808 nm; power = 1 W) from http://www.freaklasers.com/ for 30 s. This laser introduces much greater power than the IR flashlight used in previous studies [16, 17]. Time-lapse imaging was recorded for 40 minutes using a confocal multiphoton microscope system (Leica TCS SP5 MP, Leica Microsystems, Bannockburn, IL, USA). Upon photoexcitation, the *S. aureus* cells stained with SYTO9 fluoresce green and those stained with propidium iodide fluoresce red. The excitation/emission of SYTO9 and propidium iodide occur at 480/500 nm and 490/645 nm, respectively. The survival/necrosis rate of the microorganism was determined by using the imaging software called ImageJ (image processing and analysis in Java—National Institute of Health; http://rsbweb.nih.gov/ij/download.html). By post-confocal imaging, this software allows one to count the bacteria in order to determine the amount of live cells compared to total number of cells.

## 3. Results and Discussion

### 3.1. Characterization and Analysis of Electrospun Nanofibers

**Figure 2:**
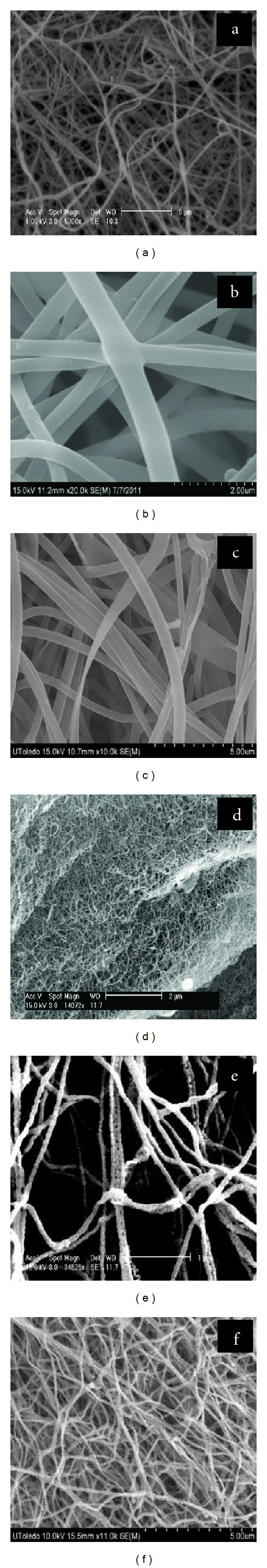
SEM images of the as-spun: (a) TiO_2_/PVP cermer, (b) iron-doped TiO_2_/PVP cermer, and (c) silver-doped TiO_2_/PVP cermer; the morphology of the nanofibers fired at 700°C for 2 h is shown in (d), (e), and (f), respectively.

The morphological features of the as-spun cermer composites of the titania, iron-doped titania, and silver-doped titania formulations are shown in (Figures 2 (a) through 2(c)). Figures 2(d), 2(e), and 2(f) show the microstructure in the calcined (700°C/2 h) pure, Fe-doped, and Ag-doped fibers, respectively.

These images ensure the quality of fibers in terms of the absence of intertwining, twisting, branching, liquid globule entrapment, and so forth. Moreover, the distinct physical structure consisting of well-formed nanofibrils as well as the difference between the as-spun and calcined fibers is clearly seen in each formulation. The decrease in fiber diameter upon calcination is also noticeable in all three specimens; approximate measurements carried out on high magnification images showed the diameter of the calcined fibers to be about 1/4 that of the as-spun fibers. Furthermore, the fibers are porous and less than 150 nm across; they are comprised of interconnected nearly monosized grains (~20–25 nm) as were deciphered by SEM and transmission electron microscopy (TEM) imaging [16]. This characteristic makes the structure breathable and, therefore, quite amenable for the intended medical application. Wong et al. [30] have demonstrated that nanosized titanium dioxide particles were more efficient at absorbing radiation than particles with larger dimensions, attributing this to the larger surface area per unit mass of the nano-sized particles as opposed to those of conventional sizes. Since, the method reported here involves photoactivation, a larger surface area (rendered by nano-sized titania fibers) would be more effective for enhanced absorption upon illumination.

Emami-Karvani and Chehrazi [31] also observed increased antibacterial activity of ZnO nanoparticles with reduction in particle sizes, against *E. coli* and *S. aureus*. Although they used ZnO, similar antibacterial response is envisaged with TiO_2_ nanoparticles as demonstrated in the present work.

The presence of dopants was confirmed by the elemental mapping (not shown) carried out by energy dispersive X-ray spectroscopy (EDS) attachment with SEM; the semi-quantitive EDS analysis gave the gross concentration (weight%) of silver and iron oxide in the two samples. Due to their low concentrations, it was rather difficult to ascertain the presence of dopants by X-ray diffraction (XRD), which was only able to reveal the presence of titania. The energy dispersive X-ray spectroscopy (EDS) attachment with SEM software also showed the formation of TiO_2_ in the nanofibers across all specimens with confirmation of the presence of individual doping elements. The XRD patterns collected on the calcined samples were identical (titania in rutile phase). The XRD signatures of pure and Fe-doped titania nanofibers were shown and discussed in detail in an earlier paper [16] as for the preferential transformation of the fibers in rutile modification.

**Figure 3:**
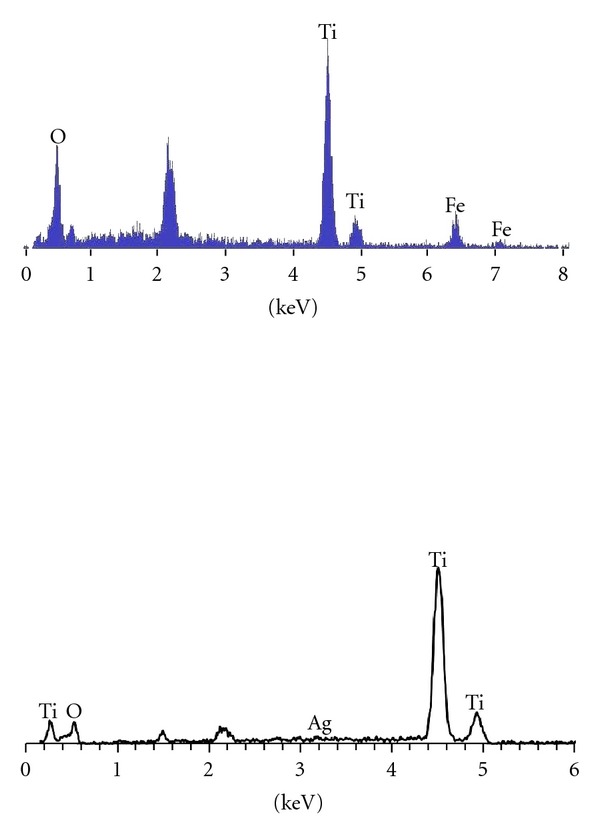
EDS spectra of the Fe-doped (top) and Ag-doped (bottom) TiO_2_ nanofibers, after calcination at 700°C/2 h.

 The as-spun fibers are a cermer (ceramic-polymer) composite. This accounts for the larger diameters seen in the as-spun fibers in Figure 2. Substantial decrease in the fiber dimensions is expected upon calcination, as the firing process removes the polymer components in the form of carbon dioxide and water mostly. The EDS signatures of the Fe- and Ag-doped TiO_2_ fibers are shown in Figure 3.

In the case of Fe-doped fibers, the EDS analysis showed iron oxide concentration to be 5 wt% [16]. The EDS analysis of the nominally 1 wt% Ag-doped TiO_2_ nanofibers gave the concentration of silver to be 1.22 wt%.

### 3.2. Bactericidal Efficacy of the Nanofibers on *S. aureus* Suspension 

**Figure 4:**
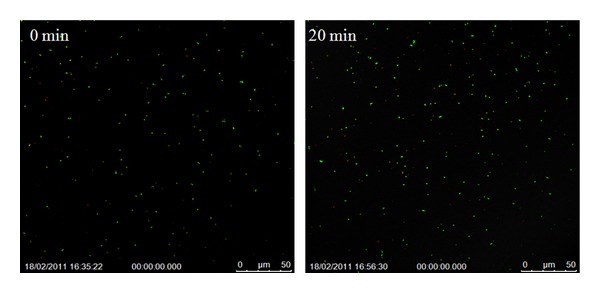
Control experiment with *S. aureus* suspension without titania or IR radiation.

With the aid of the Invitrogen live/dead assay, the cell necrosis was followed in real time and still images were taken at different intervals, starting from the time of activation (*t* = 0 min) to 40 min; the activation was caused by irradiation with a hand-held IR laser device (**λ** = 808 nm).  Figure 4 shows time-lapse shots of an *S. aureus* suspension not exposed to either photoactive titania nanomats or the IR radiation. This constituted the control experiment and demonstrates the complementary function of the photoactivating substrate and radiation source to cause the desired cell necrosis.

**Figure 5:**
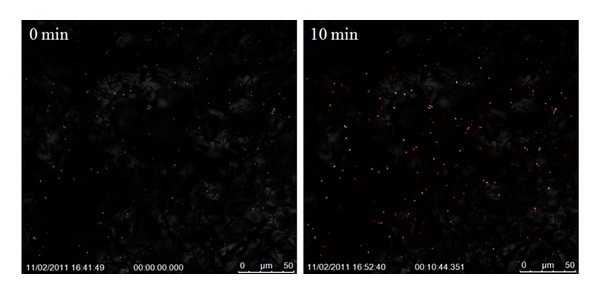
Confocal images of the bacterial colonies in pure TiO_2_ suspension in *S. aureus* broth at different times after activation by IR laser for 30 s.

**Figure 6:**
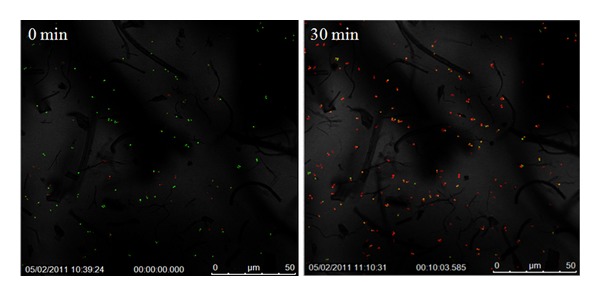
Confocal images of the bacterial colonies in Fe-doped TiO_2_ suspension in *S. aureus *broth at different times after activation by IR laser for 30 s.

**Figure 7:**
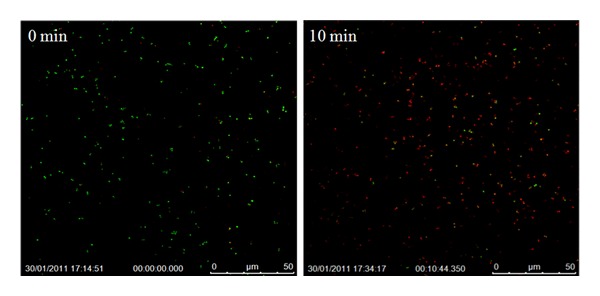
Confocal images of the bacterial colonies in Ag-doped TiO_2_ suspension in *S. aureus* broth at different times after activation by IR laser for 30 s.

The bactericidal efficacy of the pure and doped titania nanofibers towards *S. aureus* upon activation is presented in Figures 5 to 7; live cells are identified by green pixels and the dead cells by red pixels. These images clearly show the dramatic and quantitative change in the cell concentration (dead versus live) over time after exposure to activated fibers.

Figure 5 shows the influence of pure TiO_2_ nanofibers on *S. aureus* cell necrosis in a span of 10 min.

The image captured at the very induction of activation (*t* = 0 min) shows almost 100% of the cells becoming engulfed by the SYTO9 stain confirming cell viability (green pixels), while after the lapse of 10 min, the majority of cells are seen to have been disrupted by the PI stain, which penetrates the cell membrane after cells lose viability (red pixels). 

With ImageJ software, cells were counted individually at the two time intervals. For this, the still shots taken during time-lapse imaging were uploaded into the software and configured in order for all cells to be visible and countable. Cell counts and the percentage of live cells versus dead cells are given in Table 1 along with the concentration of surviving cells. As seen from Figure 5 and Table 1, the quick effect of the activated fibers with 12.8% survival rate for the *S. aureus* cells after only 10 min. could be easily discerned. After the lapse of 30 minutes since the exposure to IR photoactive pure TiO_2_ nanofibers, the cell necrosis was found to be more than 90% with only 2.5% of the cells surviving.

Figure 6 clearly shows the photoactivation-mediated bactericidal attributes of the Fe-doped TiO_2_ fibers. Even after a lapse of 30 min, the PI continues to actively penetrate the diaphragm of the* S. aureus* cells which were still viable since the time of photoactivation. The bactericidal efficacy of the Fe-doped fibers also approached 90%, with approximately 13.4% of the cells surviving after 30 min. of exposure, as seen from Figure 6 and Table 1. It should, however, be pointed out that the necrosis rate of Fe-doped fibers was lower than that of pure as well as Ag-doped TiO_2_ nanofibers, after a10 minute lapse time; the survival rate in this case was about 40%. 

**Figure 8:**
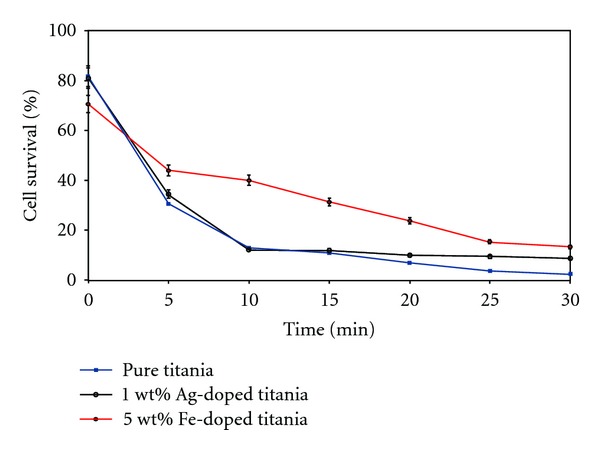
Variation in bacterial survival rates with time lapse for different fiber composition (SD: 5%).

The 1 wt% Ag-doped TiO_2_ formulation also showed high cell necrosis over the time-lapse duration, with only 12.2% and 8.9% cells surviving after 10 and 30 min, respectively, from exposure.  Figure 7 shows the still shots taken after zero and 10 min. Even though the survival rate at the end of 30 min. was quite low, it should be mentioned that the Ag-doped fibers caused necrosis of cells within as little as 10 min. with the survival rate only decreasing by 3.3% between 10 to 30 min, whereas, the pure titania fibers created a decrease of over 10.3% between these two time points. The systematic variation in the cell survival rate as a function of time after irradiation by IR laser for 30 s, is shown in Figure 8. This illustration clearly brings out the superiority of pure titania over its doped analogs.

Although the cell necrosis was seen throughout the entire bacterial suspension, it was observed more prominently in the area where the activated fibers were placed and even more specifically where the IR laser beam was most centralized and concentrated. It was this area where the time-lapse imaging was concentrated for 30 min. from the time of activation (*t* = 0 min.). The variation seen in the number of cells at different imaging time intervals is a result of cells moving in and out of the plane that is being imaged.

In comparing the effectiveness of the titania nanofibers activated by IR radiation in the present work with that of titania powder with UV radiations used by others, it becomes amply evident that there are great advantages in using an IR light source. Table 1 summarizes the results of the present work with titania nanofibers made via electrospinning and compares them with those reported on biocidal efficacy of titania powders and films, using UV radiation and other types of microorganisms.

For instance, the assessment of bactericidal effects of TiO_2_ particles irradiated by UV light has been examined by researchers to control bacterial infections in clinical applications. Azad et al. [16] have recently reported the bactericidal aspects of the electrospun titania nanofibers using multiphotons and IR flashlight with much lower power output in broths containing *E. coli*. In comparison, the advantage of the IR laser is self-evident in terms of greater cell death in the case of more resistant bacterial species, in this work. It is worth pointing out that as related to the clinical relevance of this study, *S. aureus* is the main bacterial species causing surgical site infections, which is the cause of highest mortality rate [32–34]. For example, Kneifel and Leonhardt [34] reported on the viability of various bacterial species against varying antibiotic treatments, showing increased resistance of *S. aureus* over several other microorganisms at certain concentrations of antibiotics.

From the foregoing discussion, Fe- and Ag-doped titania fibers seem comparable in their bactericidal efficacy, with plain fibers showing a notable advantage. At the end of 30 min, the number of surviving bacterial cells was between 2.5 and 13.4%. 

It is expected that the addition of dopants would increase the bactericidal efficacy of the nanofibers upon photoactivation. The advantage of one dopant over the other was not hypothesized because both Fe and Ag are known to possess excellent antibacterial properties. The results showed that Fe-doped fibers needed somewhat longer time to obtain efficacy comparable to that of pure and/or Ag-doped TiO_2_ fibers. The observed discrepancy could be ascribed to a number of factors. For example, it is likely that fibers were more concentrated in one location during one test as opposed to the other (a not easily controllable experimental artifact). This would cause greater/quicker necrosis in one instance compared to other.

On the other hand, Koseki et al. [6] used a suspension of *Staphylococcus aureus* of concentration 1 × 10^5^ cfu/mL (colony forming units) in solution containing 19 *μ*g/mL of TiO_2_ particles and irradiated them for 1 h with UV light (1.82 mW/cm^2^) in one case and with fluorescent light (80 *μ*W/cm^2^) in the other. It was found that the bacterial survival rate decreased steadily, reaching 9.4% after exposure to UV and 10.9% after exposure to fluorescent light. Yu et al. [7] have reported the fabrication of Fe-doped TiO_2_ films on stainless steel substrates by dip coating followed by calcination and their use as antibacterial agents for sterilization against *Bacillus pumilus*. In this case, a suspension of *Bacillus pumilus* of concentration 1 × 10^7^ cfu/mL (colony forming units) was placed onto the TiO_2_-coated stainless steel plate which was irradiated by a UV lamp (intensity rating 630 *μ*W/cm^2^; **λ** = 365 nm). Their results showed that the active *Bacillus pumilus* colonies on the titania films subjected to UV illumination decreased by 50% after 2 h of exposure.

 In their work, Oka et al. [8] studied the inhibition of bacterial colonization of methicillin-resistant *Staphylococcus aureus* (MRSA) suspensions (1 × 10^8^ cfu/mL) on TiO_2_ photocatalytic film prepared by direct oxidization of pure titanium substrate. In this case, the titania coating on Ti was created by etching the latter with 5–10% HF solution followed by soaking in aqueous H_2_O_2_ for 2 days. The MRSA suspension on the implant was exposed to the ultraviolet A (UVA) light for 60 min. and the number of colonizing bacteria was estimated. The bactericidal ability of the photocatalyst became apparent after 60 min, when the bacteria had almost disappeared; only about 7% bacteria were found alive. The number of colonizing bacteria on photocatalytic pins also decreased significantly *in vivo* as well. The titania film was found to be quite effective even against resistant bacterial colonization.

Recently, Hwang et al. [9] have claimed increased bactericidal activity of composite TiO_2_-ZnO nanofibers against *E. coli* and *S. aureus*. The authors show the results with and without UV irradiation and maintain that ZnO is responsible for the cell necrosis in the second case, via the formation of hydroxyl radicals. The survival rate of the bacteria in dark was less than 50%, whereas upon exposure to UV light (**λ** = 312 nm, 6 W) for 30 s, less than 15% survived. The effective inhibition was 86.7% for the composite as compared to 56.2 and 68.3%, respectively, when pure TiO_2_ and ZnO fibers were employed. However, the operative mechanism in the absence of light has not been elaborated. 

It is well known that UV radiation has higher photon energy than its IR counterpart. Therefore, from physics point of view, the higher efficacy of IR in our case seems counter-intuitive. However, calculations based on the intensity of the incident beam and the time of exposure in each case show that it is the number of photons incident per unit area during the exposure time that is key to the observed phenomenon; it is higher in the case of the present work using IR beam. This in turn, translates into higher efficiency of the IR light compared to UV by several orders of magnitude (in terms of photons/cm^2^; Table 1), since there are more photons for the time duration in comparison to the UV. This explains why the IR method used in this work for relative shorter period was more effective than the longer exposure by UV in the cases reported in the literature. We have not come across any report where the demonstration of the efficacy of IR light towards infection mitigation in terms of biocidal activity of *S. aureus* has been reported. Since Hwang et al. [9] employed a rather high wattage-light source, even for 30 s, the number of photons generated per unit area is calculated to be 2.8 × 10^20^ (Table 1), which is of the same order of magnitude as obtained in the present work and thus explains their effectiveness.

A pilot study [35] pertaining to the toxicity aspects of titania-based nanofibers concluded the lack of inducement of inflammatory responses in human cells upon exposure to infrared-activated titania nanofibers. 

Doping of titania at low levels retained bactericidal properties of the fibers. However, pure TiO_2_ nanofibers caused the most accelerated cell necrosis. The results show that pure fibers had the best characteristics overall, with a low bacterial survival rate at the end of 30 min, in addition to a faster rate of cell necrosis, which exceeded that of Ag-doped fibers at the end of 10 min. 

Reproducible experiments will undoubtedly be needed in order to justify the possibility of clinical applications. The comparison of the behavior of 1 wt% Ag-doped titania fibers versus pure titania could not be used as the ultimate conclusion that pure titania behaves better than the doped analog. It is very much likely that higher loading of silver might change this. Moreover, the results reported in this paper are only the first-stage experimentation to identify the prospect of using these formulations in clinical applications.

The main advantage, obviously, lies in the shortness of exposure time when using IR radiation (30 s) compared to UV (60 min); this would be translated into saving enormous exposure time in the practical use of these materials *in vivo*. Even though these studies are only preliminary, higher rate of cell necrosis suggests greater promise in clinical applications, where time is very critical. This is particularly true when dealing with surgical procedures and the onset of infection. More work is needed to achieve this.

## 4. Conclusion

Pure and doped TiO_2_ nanofibers were fabricated via electrospinning and thermal processing of suitable titanium (IV), Fe (III), and Ag (I) precursors with ethanolic PVP solution. The photoactivation-assisted antibacterial response of these self-standing fibrous materials was exploited by irradiating them with IR laser light for 30 s and placing them in a BSL2 gram-positive *S. aureus* bacterial suspension. The confocal microscopic results demonstrated that almost complete bactericidal activities were observed with IR exposure for a far shorter duration (30 s) with pristine as well as 5 wt% iron- and 1 wt% silver-doped titania nanofibers. The extraordinary capability of these nanofibers to eliminate *S. aureus* buildup and reduce their ability to proliferate and cause further damage to human tissue was demonstrated.

The aspect of the longevity of their effectiveness will be investigated in order to gain insight into how long these TiO_2_ fibers would continue to induce cell necrosis, once photoactivated.

## Figures and Tables

**Table 1:** Summary of results on the biocidal efficacy of titania-based systems.

Material (TiO_2_)	Exposure time	Light source	Survival rate (%)		Surviving cell concentration (#/mL)*	photon/area^‡^	Reference
Pure nanofibers	30 s	IR (*λ* = 808 nm, 5 W/cm^2^)	12.8^++^	2.5**	1.98 × 10^6^	6.1 × 10^20^	This work
5 wt% Fe-doped nanofibers	30 s	IR (*λ* = 808 nm, 5 W/cm^2^)	40^++^	9.7**	7.69 × 10^6^	6.1 × 10^20^	This work
1 wt% Ag-doped nanofibers	30 s	IR (*λ* = 808 nm, 5 W/cm^2^)	12.2^++^	8.9**	7.06 × 10^6^	6.1 × 10^20^	This work
Powder	60 min.	UV (*λ* = 352 nm, 1.8 mW/cm^2^)	9.4^+^		—	1.2 × 10^19^	[6]
Powder	60 min.	Fluorescent (*λ* = 352 nm, 0.8 mW/cm^2^)	10.9^+^		—	5.1 × 10^17^	[6]
Film on substrate	120 min.	UV (*λ* = 365 nm, 630 mW/cm^2^)	50*		—	8.3 × 10^18^	[7]
Powder	60 min.	UVA (*λ* = 365 nm, 1.1 mW/cm^2^)	7^+^		—	7.3 × 10^18^	[8]
ZnO/TiO_2_ nanocomposite	30 s	UV (*λ* = 312 nm, 6 W/cm^2^)	13.3**		—	2.8 × 10^20^	[9]

^‡^Incident photons per unit area integrated over the exposure time; it is obtained by multiplying the ratio of the source intensity (W/cm^2^) and photon energy (J) with the time of exposure (s).

*Total *S. aureus* concentration per experiment = 7.93 × 10^7^ cells/mL.

^++^10 min. time lapse after exposure.

**30 min. time lapse after exposure.

^+^60 min. time lapse during exposure.

*120 min. time lapse during exposure; *Bacillus pumilus*.
